# Reduced restenosis and enhanced re-endothelialization of functional biodegradable vascular scaffolds by everolimus and magnesium hydroxide

**DOI:** 10.1186/s40824-022-00334-x

**Published:** 2022-12-21

**Authors:** Seung-Woon Baek, Da-Seul Kim, Duck Hyun Song, Han Byul Kim, Semi Lee, Jun Hyuk Kim, Jun-Kyu Lee, Young Joon Hong, Chun Gwon Park, Dong Keun Han

**Affiliations:** 1 Department of Biomedical Science, CHA University, 335 Pangyo-Ro, Bundang-Gu, Seongnam-Si, Gyeonggi 13488 Korea; 2 Department of Biomedical Engineering, SKKU Institute for Convergence, Sungkyunkwan University (SKKU), 2066 Seobu-ro, Jangan-gu, Suwon-si, Gyeonggi 16419 Korea; 3 Department of Intelligent Precision Healthcare Convergence, SKKU Institute for Convergence, Sungkyunkwan University, 2066 Seobu-ro, Jangan-gu, Suwon-si, Gyeonggi 16419 Korea; 4 School of Integrative Engineering, Chung-Ang University, 84 Heukseok-ro, Dongjak-gu, Seoul, 06974 Korea; 5 The Cardiovascular Convergence Research Center of Chonnam, National University Hospital Designated By Korea Ministry of Health and Welfare, 42 Jebong-ro, Dong-gu, Gwangju, 61469 Korea; 6 Division of Cardiology of Chonnam, Cardiovascular Convergence Research Center Nominated By Korea Ministry of Health and Welfare, National University Hospital, 42 Jebong-ro, Dong-gu, Gwangju, 61469 Korea

**Keywords:** Cardiovascular disease, Biodegradable vascular scaffold, Everolimus, Magnesium hydroxide, Restenosis, Re-endothelialization, Inflammation

## Abstract

**Background:**

Coronary artery disease is a cardiovascular disease with a high mortality and mortality rate in modern society. Vascular stent insertion to restore blood flow is essential to treat this disease. A fully biodegradable vascular scaffold (BVS) is a vascular poly (L-lactic acid) (PLLA) stent that is receiving growing interest as this is biodegradable in the body and does not require secondary removal surgery. However, acidic byproducts composed of PLLA produced during the biodegradation of the BVS can induce an inflammatory response. Magnesium hydroxide, a basic inorganic particle, neutralizes the acidic byproducts of PLLA.

**Methods:**

In this study, we investigated using a BVS coated with everolimus and surface-modified magnesium hydroxide that suppresses smooth muscle cell proliferation and protects endothelial cells, respectively. The various characteristics of the functional stent were evaluated using in vitro and in vivo analyses.

**Results:**

The BVS was successfully prepared with evenly coated everolimus and surface-modified magnesium hydroxide. A neutral pH value was maintained by magnesium hydroxide during degradation, and everolimus was released for one month. The coated BVS effectively inhibited protein adsorption and platelet adhesion, demonstrating excellent blood compatibility. In vitro analysis showed that BVS protects endothelial cells with magnesium hydroxide and selectively inhibits smooth muscle cell proliferation via everolimus treatment. The functional BVS was inserted into porcine coronary arteries for 28 days, and the results demonstrated that the restenosis and inflammation greatly decreased and re-endothelialization was enhanced as compared to others.

**Conclusions:**

This study provides new insights into the design of drug-incorporated BVS stent for coronary artery disease.

**Graphical Abstract:**

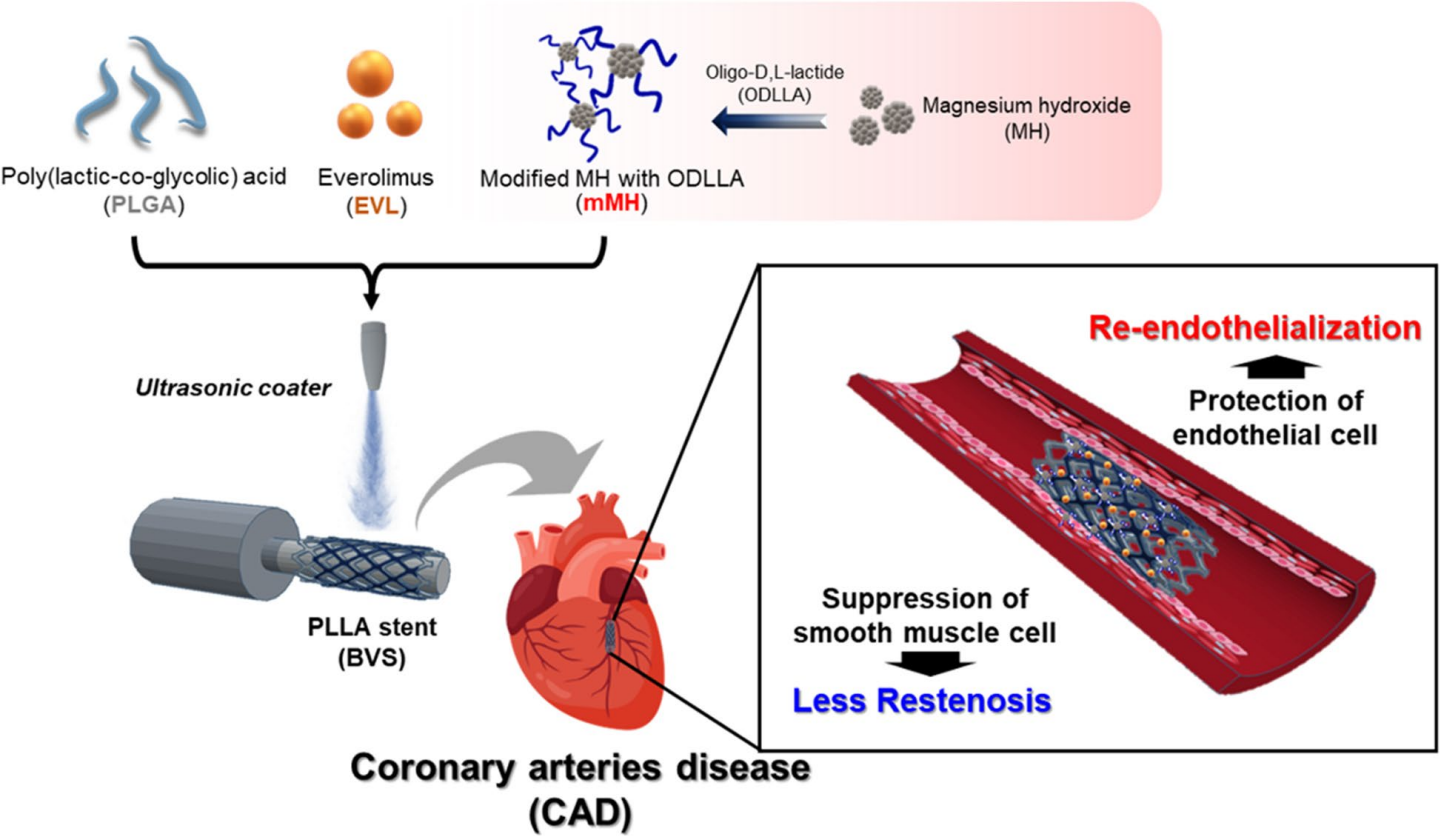

**Supplementary Information:**

The online version contains supplementary material available at 10.1186/s40824-022-00334-x.

## Background

Cardiovascular diseases, including coronary artery diseases (CADs), are increasing in incidence globally and are the leading causes of death and disability in modern society. To treat these lethal diseases, the vascular stent has been commonly used to restore blood flow. First-generation bare metal stents (BMSs), consisting of cobalt-chromium (Co-Cr) and stainless steel support the lining of the blood vessels with high mechanical properties but have been frequently reported to induce in stent restenosis (ISR) and thrombosis due to foreign-body reactions [1–3]. Second-generation drug-eluting stents (DESs) have been developed from BMSs to overcome this problem and provide additional drug release. The coated drugs, such as anti-mitotic agents, reduced the proliferation of smooth muscle cells, decreasing the incidence of ISR to 60%–80% [1, 4]. However, since both BMS and DES are permanently implanted that they cannot be removed and replaced, and the corrosion of metals and release of toxic metal ions causes late thrombosis, inflammatory response, and ISR [5]. These problems result in stent insertion failure and death of the patient in clinically [6]. In recent studies, a biodegradable vascular scaffold (BVS) has been adopted to solve the previous issues of BMS and DES. The biodegradable poly (L-lactic acid) (PLLA) is fully absorbed in the body and has consequently received much attention because secondary removal surgery is unnecessary. PLLA, which has been approved by the FDA, is an aliphatic polyester with excellent biocompatibility, biodegradability, and mechanical properties that has been used in medical and pharmaceutical fields [7]. The PLLA-based Absorb™ BVS (Abbott Vascular; CA, USA) was a pioneer of commercialized biodegradable coronary stents. The Absorb™ BVS consisted of a 7-μm-thick coating layer of drug (everolimus, EVL) on a 150-μm-thick PLLA scaffold core. Unfortunately, randomized clinical trials and long-term follow-up of observational registration studies identified clinical events, including local inflammation and thrombosis that were caused by scaffold degradation, leading to withdrawal from the clinical trials and the market [8, 9]. We hypothesized that one of the pivotal reasons for this failure was an acidic byproduct of PLLA, lactic acid, which induced an acidic microenvironment at the implanting site. It also has been many pointed out as an inflammatory response inducer associated with neointima formation, ISR, and thrombosis [10–12]. Although acidic byproducts of PLLA are oxidized to pyruvate and then metabolized and eliminated to water and carbon dioxide in the TCA cycle in the mitochondria, small amounts of acid are gradually accumulated at the implantation site. Consequently, an acidic environment would stimulate an inflammatory response [5, 13–15].

Magnesium hydroxide [Mg(OH)_2_, MH], a basic inorganic particle, has been reported to neutralize acidic byproducts of aliphatic polyester-based scaffolds and inhibit the inflammatory response [16, 17]. As a constituent of antacids, MH is dissolved partially in water to produce magnesium and hydroxide ions and performs neutralization by combining with acidic H^+^ ions. Furthermore, magnesium ions (Mg^2+^) released from MH can maintain the homeostasis of vascular endothelial cells, which protects blood vessels from various stimuli [13]. In our previous studies, we fabricated a surface-modification of MH with hydrophobic materials, such as fatty acids and oligomers, to improve dispersion stability in organic solvents and the interaction between hydrophilic MH and hydrophobic polymer matrix. Moreover, the effects of pH neutralization and inhibition of inflammatory response in vitro and in vivo were investigated [18–22].

Rapamycin, also known as sirolimus or EVL, is a macrocyclic lactone produced by *Streptomyces hygroscopicus* and is one of the most used drugs for DES and can inhibit restenosis [23]. Rapamycin suppresses the phosphoinositide-3-kinase/Akt/mammalian target of rapamycin (PI3K/Akt/mTOR) pathway. The serine/threonine protein kinase Akt, also known as protein kinase B, is a substrate of mTOR signaling in mammalian cells and promotes cell proliferation, growth, metabolism, and autophagy [24]. Akt binds to the FKBP12 protein, and this complex allosterically inhibits the assemblage of mTORC1 substrates by directly binding to the FKBP12-rapamycin-binding (FRB) domain [25]. There are various approaches to ameliorate the function of stents through a coating system. Kim et al. investigated the inhibitory ability of stents on smooth muscle cells with diverse types of coated rapamycin [23]. Shen et al*.* developed biodegradable coronary stents, including heparin, using a 3D printer [5]. These studies effectively inhibited the proliferation of smooth muscle cells. However, endothelial cells, which are essential for vascular regeneration, can also be inhibited because rapamycin affects all types of cells.

In previous studies, we developed a Co-Cr BMS with rapamycin and MH. The coated BMS was implanted in porcine coronary arteries and exhibited excellent biocompatibility with re-endothelialization and anti-inflammatory effect [26, 27]. In this study, we prepared surface-modified MH (mMH) with oligo-D,L-lactide (ODLLA) and then made functional PLLA BVS coated with EVL and mMH (BVS/EVL/mMH) to inhibit restenosis and inflammation and protect endothelial cells. We demonstrate that the proliferation and migration of smooth muscle cells were reduced by the inhibitory effect of EVL on the mTOR pathway and that mMH protected the endothelial cells from EVL-induced damage. The capacity of this system for inhibiting inflammatory response and restenosis and enhancing re-endothelialization was investigated in vitro and in vivo.

## Materials and methods

### Materials

Biodegradable vascular stent (BVS; Poly-L-lactide, PLLA) and everolimus (EVL) were provided by CGBIO (Korea). Poly(D,L-lactide-co-glycolide) (PLGA, RG 505, Mw = 110 kDa) was obtained from Evonik Industries AG (Germany). Magnesium hydroxide, stannous octoate, albumin from human serum, and fibrinogen from human plasma were purchased from Sigma–Aldrich (MO, USA). Chloroform and tetrahydrofuran (THF) were purchased from Daejung Co. Ltd. (Korea). D,L-Lactide was obtained from Junsei Chemical (Japan). Proteinase K and Universal RNA Extraction Kit were purchased from Bioneer (Korea). Platelets were obtained from the blood center of the Korean National Red Cross (Korea). A 20% SDS solution was purchased from Bio-Rad (CA, USA). Human coronary artery endothelial cells (HCAECs), human coronary artery smooth muscle cells (HCASMCs), endothelial cell growth medium-2 (EGM-2), and smooth muscle cell growth medium (SmGM) were purchased from Lonza (Switzerlan). Dulbecco’s phosphate buffered saline (DPBS) solution was obtained from WELGENE (Korea). A cell-counting kit (CCK-8) was obtained from Dongin LS (Korea). The live/dead staining kit (Calcein AM/EthD-1), nuclease-free water, and lipophilic trace DiO was supplied from Invitrogen (MA, USA). 2’,7’-Dichlorodihydrofluorescein (DCF-DA) was provided by Cayman Chemical (MI, USA). Yorkshire × Landrace F1 crossbred castrated male pigs were supplied in the laboratory animal center of Chonnam National University Medical Institute for 5–10 days prior to the experiment. All chemicals were laboratory reagent grade and used without purification.

### Synthesis and characterization of mMH

The ODLLA-grafted MH particles (mMH) were synthesized as previously described [19, 28]. The reaction was initiated by mixing stannous octoate (1/10,000 mol ratio of D,L-lactide), 1 g MH, and 1 g D,L-lactide. The polymerization process was conducted under a vacuum for 24 h at 150 °C. Reaction products were washed with chloroform: acetone (6:4) and centrifugated at 7,000 rpm. Reaction products were then vacuum-dried for 72 h at 25 °C.

The mMH was visualized using transmission electron microscopy (TEM; Hitachi, Japan). The chemical binding and grafting ratio of mMH was evaluated by attenuated total reflection-Fourier transform infrared (ATR-FTIR; PerkinElmer, MA, USA) and thermogravimetric analyzer (TGA; PerkinElmer, MA, USA), respectively. The size distribution of mMH was investigated using dynamic laser scattering (DLS; Malvern Panalytical, England), The pH titration assay was investigated by adding a certain amount of mMH to 12 mM L-lactic acid as a degradation product of PLLA and measuring the pH using a digital pH-meter (Orion Star A211 pH Benchtop Meter, Mettler Toledo, OH, USA).

### Preparation and characterization of the BVS/EVL/mMH

BVS was coated using an ultrasonic spray coater (Noanix, Korea). The coating solution was prepared by dissolving 3.7 mg/mL EVL with 2 mg/mL PLGA and also further dispersing 0.15% mMH in THF to obtain BVS/EVL and BVS/EVL/mMH, respectively.

The surface and element distribution of the BVS/EVL/mMH were visualized via field emission scanning electron microscopy (FE-SEM, Hitachi, Japan) and energy dispersive spectroscopy (EDS). To confirm the coated mMH and EVL, FTIR and X-ray diffractometer (XRD; D2 phaser, Bruker, Germany) was used.

The loading amount of EVL was measured by high-performance liquid chromatography (HPLC; Thermo Scientific Inc, MA, USA). The mobile phase consisted of water, acetonitrile, and methanol; the flow rate was 1.00 mL/min. The quantity of emitted EVL was measured for 10 min at 278 nm. The loading amount of mMH was investigated using inductively coupled plasma optical emission spectrometer (ICP-OES; Optima 8000, PerkinElmer, MA, USA) after fully dissolving each BVS in a 5% nitric oxide solution.

Mechanical properties were evaluated by a universal testing machine (UTM, TO-101, Testone, Korea) following ASTM standard D638. Tensile strength, elongation, and Young’s modulus were measured by coating on PLLA film cut into dumbbell-shaped specimens (14 × 6 × 2 mm^3^) under the crosshead speed of 10 mm/min at 25 °C.

### Degradation behaviors and drug release profile

Each sample was immersed in 1 mL PBS solution (pH 7.4) with proteinase K (0.02 mg/mL, Bioneer, Korea) at 37 °C. The pH changes were investigated at identical times with a digital pH-meter. The solution in the coated BVS was removed and dried for 24 h under vacuum conditions and then the remaining mass was measured. The remaining weight was evaluated with the equation below, where W_AD_ (Weight after degradation) refers to the initial weight of the coated BVS and W_BD_ (Weight before degradation) refers to the weight of the coated BVS after decomposition. To evaluate MH release, the BVS-immersed PBS solution used to assess the degradation behavior was dissolved in 5% nitric acid solution. Mg element concentration of this solution was measured by ICP-OES.

$$\mathrm{Weight}\;\mathrm{loss}\;(\%)=\frac{W_{AD}}{W_{BD}}\times100$$

The drug release profile was investigated via HPLC equipped with a UV detector. The separation was performed using a Luna 3u c18 (2) 100A, LC column measuring 150 × 460 mm (Phenomenex Inc., CA, USA). The mobile phase consisted of water, acetonitrile, and methanol (5:45:40 v/v) at a flow rate of 1.00 mL/min. The amount of EVL was measured at 278 nm for 10 min.

### Protein adsorption analysis

The evaluation of protein adsorption on the sample was conducted with human plasma fibrinogen and albumin. BVS, BVS/EVL, and BVS/EVL/mMH were placed in PBS solution at 37 °C for 30 min for hydration. Fibrinogen (0.2 mg/mL) and albumin (3 mg/mL) solution was used to treat the samples at 37 °C for 1 h, respectively. After removing each solution, the samples were rinsed three times with distilled water. To elute proteins, SDS (5%) was added and incubated overnight at 37 °C. The protein concentration in each well was evaluated with a micro-BCA kit following the manufacturer’s instruction. Measurement of the absorbance was performed at 562 nm.

### Platelet adhesion analysis

The coated BVS was sterilized for 10 min under UV light, and samples were hydrated for 1 h in 1 mL of PBS solution. Platelet solution (5 × 10^4^ platelets/uL) as prepared with platelet-rich plasma and platelet-poor plasma. The BVS was immersed with 1 mL of the platelet solution and incubated for 2 h at 37 °C. The BVS was then cleaned three times using PBS solution. To observe SEM images of adherent platelets, the samples were fixed in 2.5% glutaraldehyde solution for 1 h. These were successively dehydrated in 50, 60, 70, 80, 90, and 100% ethanol. To quantify adhered platelets, 2% Triton X-100 solution was added for 15 min to lyse platelets, and the lysate solution was tested for lactose dehydrogenase (LDH) activity. The LDH test was performed following the manufacturer’s protocol (MK401; Takara, Kusatsu, Japan).

### Cell proliferation assay

HCAECs and HCASMCs less than passage 7 were grown in a T75 tissue culture flask with 13 mL of either EGM-2 or SmGM, respectively. HCAECs and HCASMCs, were grown in an incubator with a humidified environment and 5% CO_2_ at 37 °C and were then seeded at 1 × 10^5^ cells/mL on each BVS, respectively. After 24 h, any medium was removed, and 400 μL of 10% CCK-8 solution was added to each well in the dark. The absorbance was then measured after 2 h incubation using a SpectraMax M2 plate reader (Molecular Devices, CA, USA) at 450 nm.

### Wound healing assay

HCAECs were seeded into 6-well plate at a density of 1.5 × 10^5^ cells per well. A sterile 1-mL pipette tip was used to scratch the monolayers once the cells had reached 100% confluence. And each BVS was treated with indirectly co-cultured system using the trans-well inserts (SPLInsert™, 37,524, SPL, Korea). Plates were incubated at 37 °C in 5% CO_2_ for 9 h. Cell migration was measured using Image J (ImageJ 1.44p, National Institutes of Health Bethesda, MD, USA).

### Antioxidant effect

HCAECs were cultured in 24-well tissue culture plates and treated with conditioned media (samples were immersed for 1 d at 37 °C). Cells were incubated with 20 μM DCF-DA solution in media for 45 min at 37 °C in the dark. After washing three times with PBS solution, live and DCF-DA positive cells were visualized using a fluorescent microscopy (CKX53; Olympus, Tokyo, Japan).

### Quantitative real time PCR

qRT-PCR was conducted to examine the anti-inflammation and angiogenesis-related gene expressions in vitro*.* HCAECs and HCASMCs were cultured and treated with conditioned media for 24 h in the same way as the “Antioxidant assay” explained earlier. Following the manufacturer's instructions, AccuPrep® Universal RNA Extraction Kit (Bioneer, Korea) was used to extract the total cellular RNA from cells. cDNA was synthesized from the extracted RNA using PrimeScript RT Reagent Kit (Perfect Real Time, Takara, Japan). qRT-PCR was performed using SYBR Green PCR Master Mix (Applied Biosystems, Thermo Scientific Inc, USA) with primers (Table S1). The 18S rRNA was used as a reference gene to determine the expression of genes involved to angiogenic and inflammatory processes using the 2Ct technique.

### Animal test for BVS implantation

The Ethics Committee of Chonnam National University Medical School and Chonnam National University Hospital approved this animal study (CNUHIACUC-21013), which conformed to the Guide for the Care and Use of Laboratory Animals published by the US National Institutes of Health (Publication NO. 85–23 revised 1996). Pigs were narcotized with xylazine (3 mg/kg), tiletamine and zolazepam (2.5 mg/kg), and azaperone (6 mg/kg). An intravenous (IV) catheter was put in the marginal ear vein for administration of emergency drugs and fluids, such as epinephrine and antiarrhythmic agents (amiodarone hydrochloride). IV fluid administration of 0.9% saline was continuously maintained throughout the experiment. Pigs were intubated, and anesthesia was maintained using an inhalation anesthetic of 1% sevoflurane in the oxygen. Tramadol HCl (5 mg/kg) was administered IV pre- and post-operatively to reduce pain. The coronary artery of an 8-week-old pig was used, and angiography was used to validate the insertion of the stent. We utilized the left anterior descending and left circumflex arteries. The pigs were administered with 75 mg clopidogrel and 100 mg aspirin daily for 5 days before the procedure.

### Optical coherence tomography analysis

For neointima of pig blood vessels, the carotid artery was excised and measured with optical coherence tomography (OCT; Model C7Xr Dragonfly Optis Imaging Catheter, St. Jude Medical, MIN, USA). The coronary artery was fixed to the guide wire connected with a water box dedicated to in vitro experiments. An imaging catheter (the C7 Dragonfly) was inserted into the coronary artery through the guide wire. OCT images were obtained by connecting the Dragonfly Duo and the imaging catheter, and neointimal vessels were measured using Light-Lab imaging (offline review workstation).

### Histopathologic and immunohistochemical analyses

The coronary artery tissues were harvested, fixed in 4% paraformaldehyde (Wako, Japan), embedded in paraffin, and cut into 5-μm sections. Immunohistochemistry was performed using anti-alpha smooth muscle actin (α-SMA, 1:100; Abcam, Cambridge, UK), and anti-CD31 (ab108595, Abcam, USA) antibodies at a 1:100 dilution and the appropriate secondary antibodies. The tissues were stained using hematoxylin and eosin (H&E) and Carstair's stain kit.

### Statistical analysis

All statistical analyses were accomplished using GraphPad Prism (San Diego, CA, USA). One-way ANOVA with Tukey’s multiple comparison posttest was performed to compare the samples (*n* ≥ 3). Results were not considered significant (ns) when *p* > 0.05 and statistically significant when * *p* < 0.05, ** *p* < 0.01, and *** *p* < 0.001, and **** *p* < 0.0001.

## Results

### Preparation and characterization of the BVS/EVL/mMH

**Fig. 1:**
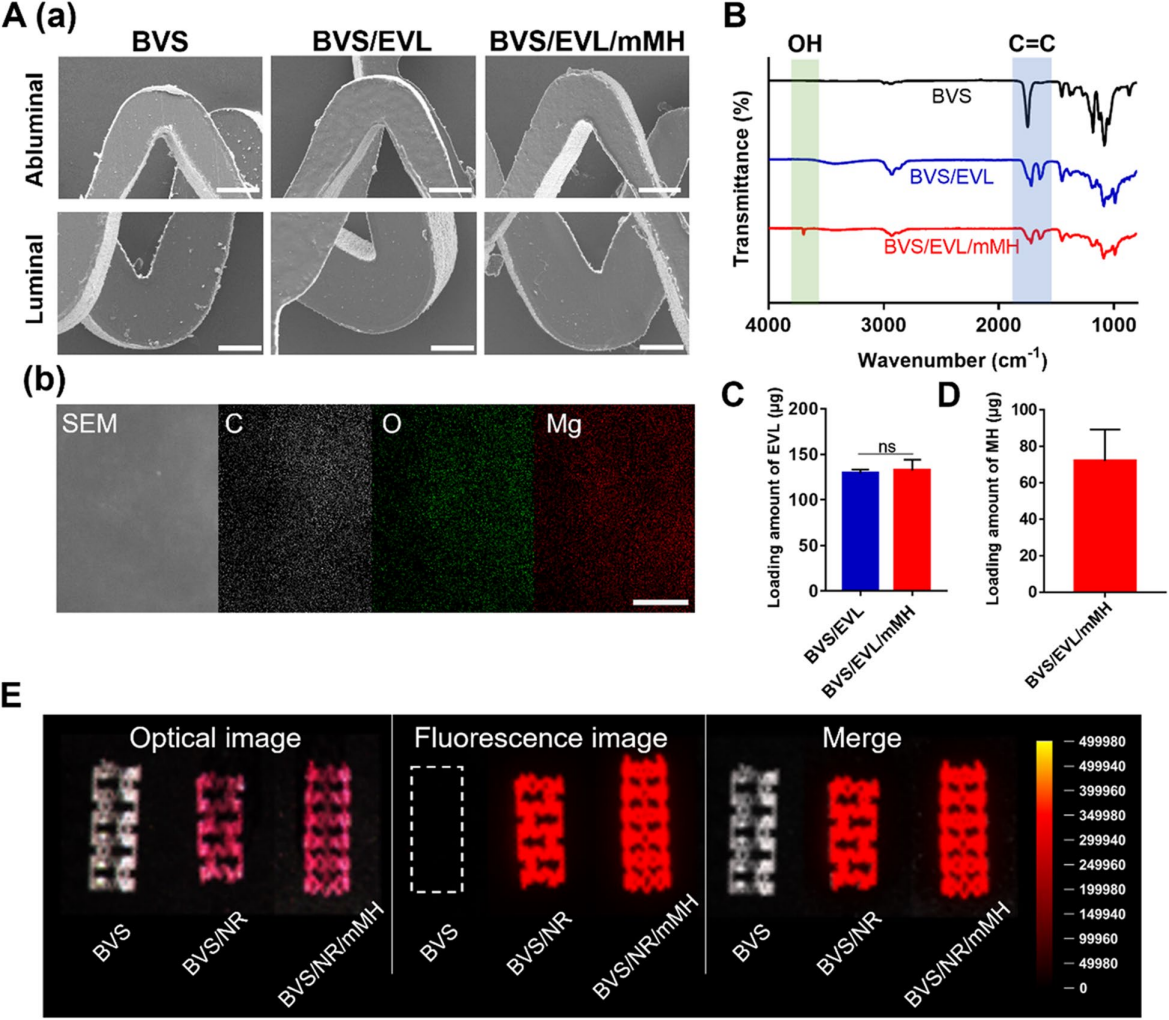
Characterization of the coated BVS. (A) SEM images of each BVS (a); scale bar indicates 200 μm (Abluminal) and 100 μm (Luminal) and EDS mapping images of C, O, and Mg elements on BVS/EVL/mMH (b); scale bar indicates 50 μm. The loading amount of (C) EVL and (D) MH in each BVS. (E) Fluorescence images using FOBI of the BVS coated with Nile Red (NR) instead of EVL

The characterization of synthesized mMH was investigated as shown in Figs. S1, S2, S3, S4, S5. The observed size of mMH was 293.5 nm based on the TEM image and size distribution. The peaks of -OH stretching vibration at 3699 cm^−1^ of unmodified MH and peaks of the carbonyl (-C = O) at 1709 cm^−1^ of ester groups in ODLLA of mMH were both displayed in ATR-FTIR spectra. The residual masses of MH and mMH from the TGA thermograms were 69% and 15%, respectively. The residual mass implied that 54% ODLLA was bound to mMH. The coating solution was prepared by dispersing mMH and dissolving EVL with PLGA in THF. Compared with the unmodified MH precipitated in THF, the mMH displayed a high dispersion stability (Fig. S6). The BVS was coated using an ultrasonic spray coater. The surface morphology and mMH distribution of coated BVS were observed using SEM/EDS. The smooth surfaces were maintained even after coating, and no intergroup differences were shown in the roughness of the BVS, BVS/EVL, or BVS/EVL/mMH (Fig. 1A(a)). The C, O, and Mg elements in the BVS/EVL/mMH were revealed in a widely distributed state, whereas only C and O elements were detected in the BVS and BVS/EVL (Figs. 1A(b) & S7). The ATR-FTIR spectra of the BVS, BVS/EVL, and BVS/EVL/mMH were presented in Fig. 1B. The peak at 1741 cm^−1^_,_ which is characteristic of EVL, was observed in the BVS/EVL and BVS/EVL/mMH. In addition, the peak at 3699 cm^−1^_,_ − OH stretching vibration of MH, was presented with BVS/EVL/mMH. The mMH contained in the BVS/EVL/mMH was measured using XRD (Fig. S8). The XRD pattern of the BVS/EVL/mMH was observed at 2θ of 18.4˚, which is the pattern of MH. The loading amount of EVL in the BVS/EVL and BVS/EVL/mMH was 129 and 132 μg, respectively, and there were no significant differences (Fig. 1C). In Fig. 1D, 71.82 μg of mMH was contained in the BVS/EVL/mMH. To indicate that stents were successfully coated in drug, BVS was coated with Nile Red (NR) instead of EVL (Fig. 1E). In the optical images, BVS/NR and BVS/NR/mMH (coated with NR) are shown in red, whereas BVS appears white. BVS did not fluoresce, although both BVS/NR and BVS/NR/mMH fluoresced red. The fluorescence intensities of the BVS/NR and BVS/NR/mMH were similar and were evenly distributed throughout the BVS. Any possible changes in mechanical properties after coating were investigated using a universal testing machine conducted after coating (Fig. S9). All samples were observed to have tensile strength, elongation, and Young’s modulus of approximately 60 MPa, 2.5%, and 4.5 GPa, respectively. This result demonstrated that the coating does not affect the mechanical properties of the stent.

### Degradation behavior and release profile of the BVS/EVL/mMH

**Fig. 2:**
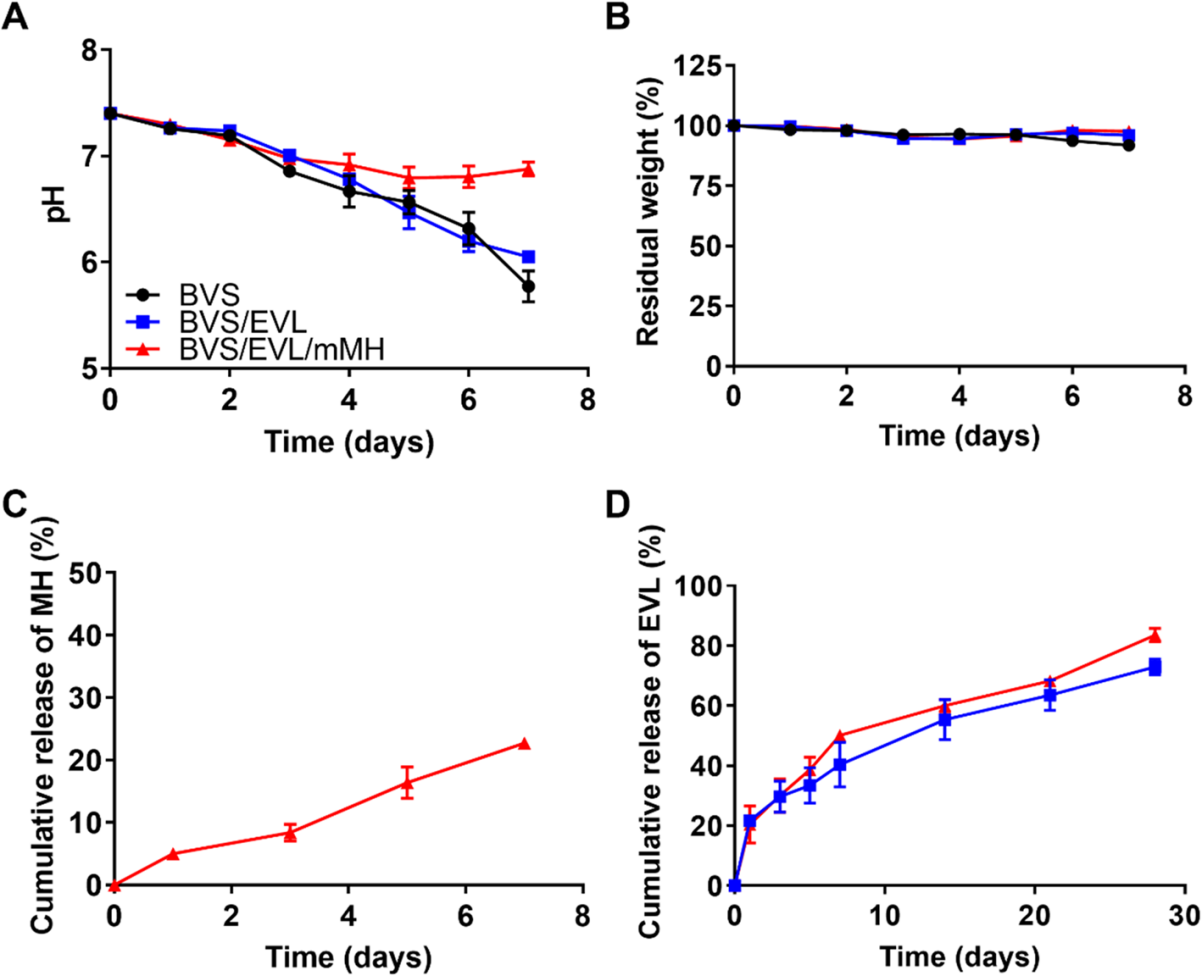
Degradation behavior and drug release profile. The changes of (**A**) pH value, (**B**) residual weight, and (**C**) cumulative release of MH during degradation in PBS solution with 0.02 mg/mL proteinase K for 7 days at 37 °C. (**D**) Cumulative release of EVL from BVS/EVL and BVS/EVL/mMH in PBS solution for 28 days at 37 °C

The degradation behavior of the coated stents was measured under accelerated conditions. The coated BVS was immersed in PBS solution with 0.02 mg/ml proteinase K at 37 °C for 7 days. The pH value of the BVS, BVS/EVL, and BVS/EVL/mMH slightly decreased to 6.86, 7.01, and 6.98, respectively, from 7.4 after 3 days. After 7 days, the pH of BVS and BVS/EVL rapidly reduced to 5.77 and 6.05, respectively, whereas that of BVS/EVL/mMH was maintained at 6.88 (Fig. 2A). There was no significant difference in the residual weight of coating between the BVS, BVS/EVL, and BVS/EVL/mMH, although this decreased to 91.8%, 96.0%, and 97.7%, respectively, after degradation for 7 days (Fig. 2B). The release of MH from BVS/EVL/mMH during degradation was measured using ICP-OES (Fig. 2C). After 3 days, 8.4% of MH was released, and this increased after 7 days to 22.73% of MH being released. The release of EVL was assessed in PBS solution at 37 °C under standard conditions, i.e., not accelerated, to measure the release rate in an environment similar to the human body (Fig. 2D). EVL from the BVS/EVL and BVS/EVL/mMH was burst-released during the initial 3 days, releasing 29.7% and 30.0%, respectively. Subsequently, 72.96% and 83.53% of EVL were released for 28 days, respectively [29, 30].

### Blood compatibility of the BVS/EVL/mMH

**Fig. 3:**
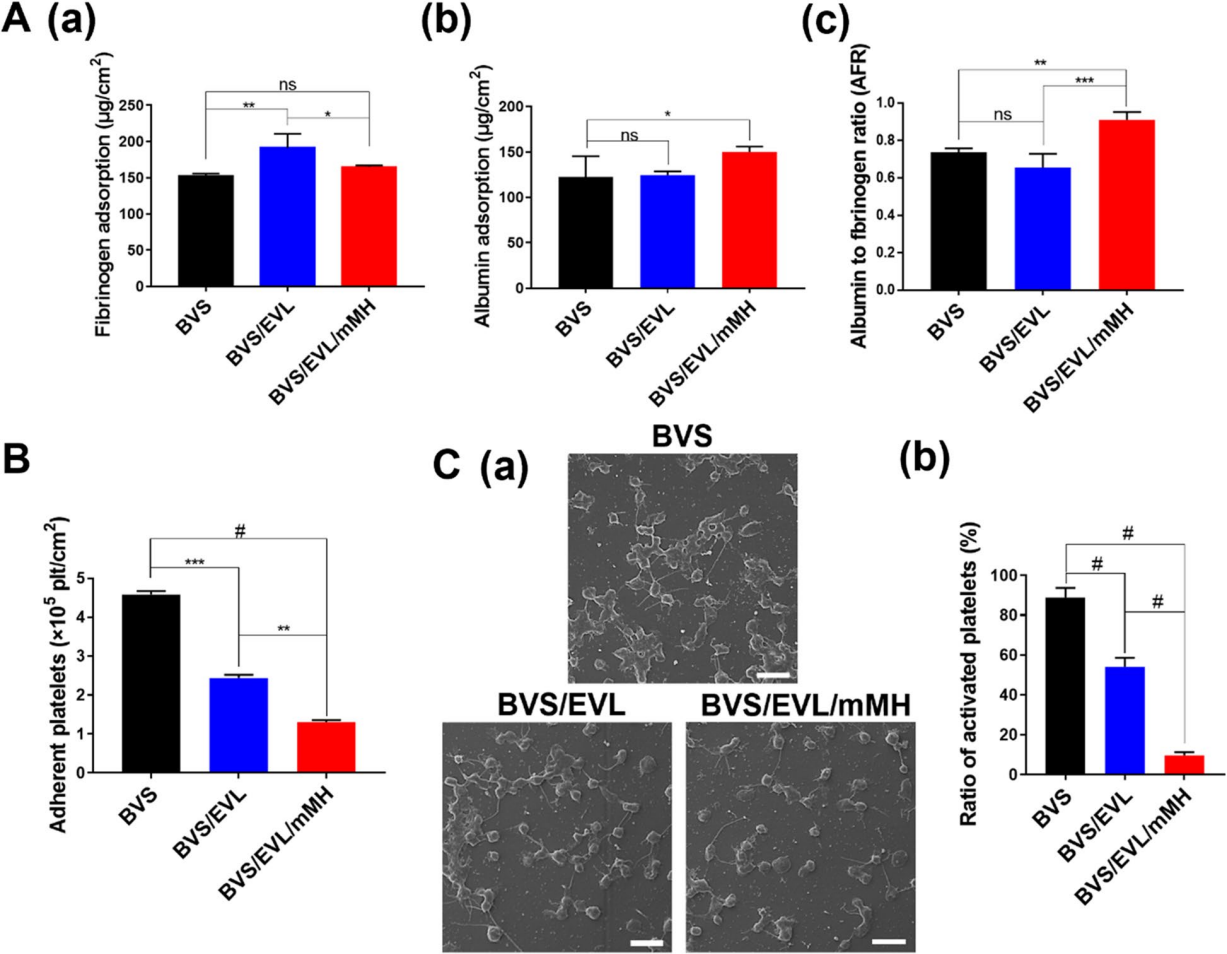
(A) Fibrinogen (a), albumin (b), and albumin to fibrinogen ratio (AFR) (c) adsorbed to the surface of each BVS. (B) The number of adhered platelets on the surface of BVS, BVS/EVL, and BVS/EVL/mMH. (C) The SEM images (a) and the ratio of activated platelets (b) of adhered platelets on the surface of each BVS. Scale bar indicates 10 μm. *p < 0.05, **p < 0.01, ***p < 0.001, and ^#^*p* < 0.0001 indicate statistically significant differences, respectively

The adsorption of fibrinogen and albumin was measured to evaluate blood compatibility. Fibrinogen was adsorbed onto the surface of the BVS, BVS/EVL, and BVS/EVL/mMH at 152.71, 183.9, and 165.54 μg/cm^2^, respectively. The amount of fibrinogen adsorbed to the BVS/EVL significantly increased compared with that adsorbed to BVS, whereas the adsorption of fibrinogen on the BVS/EVL/mMH was inhibited by MH and adsorbed in an amount similar to the BVS as shown in Fig. 3A(a). Albumin was adsorbed at 122.8, 124.64, and 150.08 μg/cm^2^ onto the surface of the BVS, BVS/EVL, and BVS/EVL/mMH, respectively. The BVS/EVL/mMH adsorbed a higher amount of albumin compared with that absorbed by either BVS or BVS/EVL, which both adsorbed the same amount (Fig. 3A(b)). The ratio of albumin to fibrinogen (AFR) adsorbed to each BVS is shown in Fig. 3A(c). The AFR values of the BVS, BVS/EVL, and BVS/EVL/mMH were 0.74, 0.65, and 0.91, respectively.

The ability of platelets to adhere onto the coated BVS and then activate is shown in Fig. 3B and C; using the LDH assay, the number of adherent platelets on coated BVS was determined as 4.58 × 10^5^, 2.43 × 10^5^, and 1.30 × 10^5^ for BVS, BVS/EVL, and BVS/EVL/mMH, respectively (Fig. 3B). Figure 3C(a) shows the number of inactivated and activated platelets obtained in SEM images. The number of adhered platelets decreased in the order of BVS, BVS/EVL, and BVS/EVL/mMH. Although platelets adhering to the surface of the BVS and BVS/EVL were observed in activated types (i.e., intermediate pseudopodal and fully spread), inactivated platelets (round, discoid, and dendritic pseudopodal) were displayed on BVS/EVL/mMH. A quantitative number of activated platelets is shown in Fig. 3C(b) The activated platelet ratio for the BVS, BVS/EVL, and BVS/EVL/mMH was 88.89%, 54.07%, and 9.63%, respectively; thus, the ratio of activated platelets of the BVS/EVL/mMH decreased by approximately tenfold than that of the BVS. As expected, in SEM images, the platelet adhesion of BVS/EVL/mMH was more than approximately three-fold lower compared with that of the BVS. The platelet adhesion with BVS/EVL was also twofold lower than that of the BVS, and the difference was doubled.

### Protection effects on endothelial cell of the BVS/EVL/mMH

**Fig. 4:**
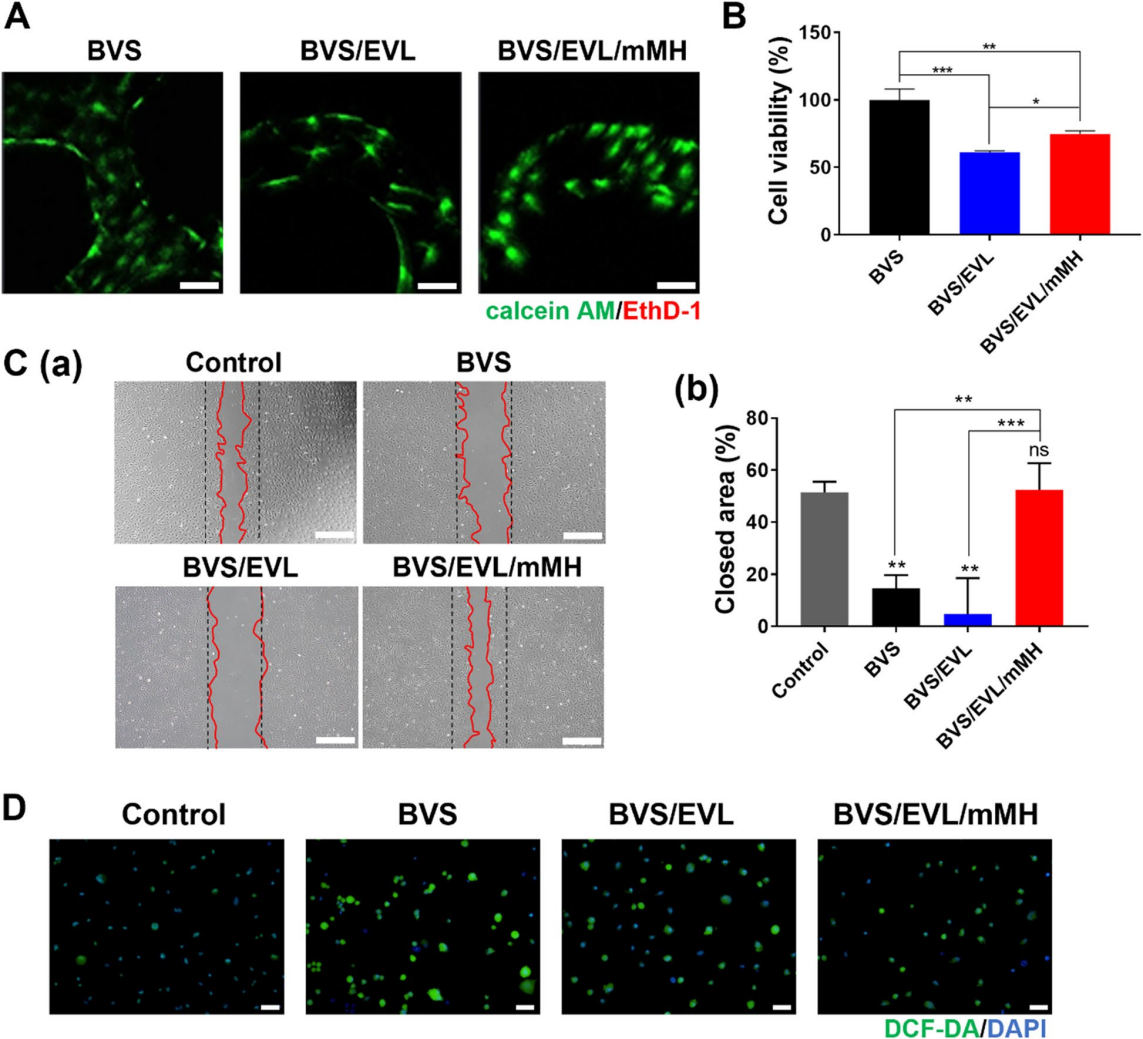
In vitro evaluation to confirm endothelial cell protection effect of the BVS/EVL/mMH. (A) Confocal microscopy images and (B) cell viability of HCAECs cultured on the coated stents at 7 days. Scale bar indicates 100 μm. (C) Optical images (a) and quantitative analysis of the closed area (b) of the scratch assay at 9 h. Scale bar indicates 500 μm. (D) Fluorescence microscopy images of DCF-DA staining in HCAECs. Scale bar indicates 50 μm. *p < 0.05, **p < 0.01, and ***p < 0.001 indicate statistically significant differences, respectively

Live/dead staining confocal images after incubating HCAECs with each group for 7 days are shown in Fig. 4A. HCAECs uniformly attached to the entirety of BVS with exhibiting high fluorescence intensity of calcein AM-positive live cells. The ethidium homodimer-1 (EthD-1) signal was rarely observed in all groups because of the well-known biocompatibility of PLLA. However, quantification using CCK-8 analysis (Fig. 4B) showed that the cell viability of the BVS/EVL groups decreased to 61.28% compared with that of the BVS control. Interestingly, the mMH incorporated group attenuated cytotoxicity of EVL by 74.68% compared with BVS. To investigate the protection effect on endothelial cells of mMH, a wound healing assay was conducted. The representative optical images and associated ratio of the closed area were measured after 9 h of scratching and sample treatment (Fig. 4C). The recovery rates of BVS and BVS/EVL were slower or rather decreased than the initial area. However, a closed area was observed with BVS/EVL/mMH that was similar to that of the control. The quantitative values for the closed area in the control and BVS/EVL/mMH had recovered by approximately 50% and were not statistically different from each other. In contrast, the closed area with BVS and BVS/EVL had only closed to 14.64% and 4.78% of the scratched area, respectively.

To demonstrate the scavenging effect of the reactive oxygen species (ROS), intercellular ROS was investigated using DCF-DA as a cell-permeant indicator (Fig. 4D) and DAPI as a nuclear counterstain. DCF-DA was oxidized to DCF by ROS to emit green fluorescence. The fluorescence image of the untreated control showed extremely weak green fluorescence. However, strong green fluorescence in the BVS and BVS/EVL was observed around the nuclei of HCAECs. The green fluorescence emitted in the BVS/EVL/mMH at a level was similar to that of the control.

**Fig. 5:**
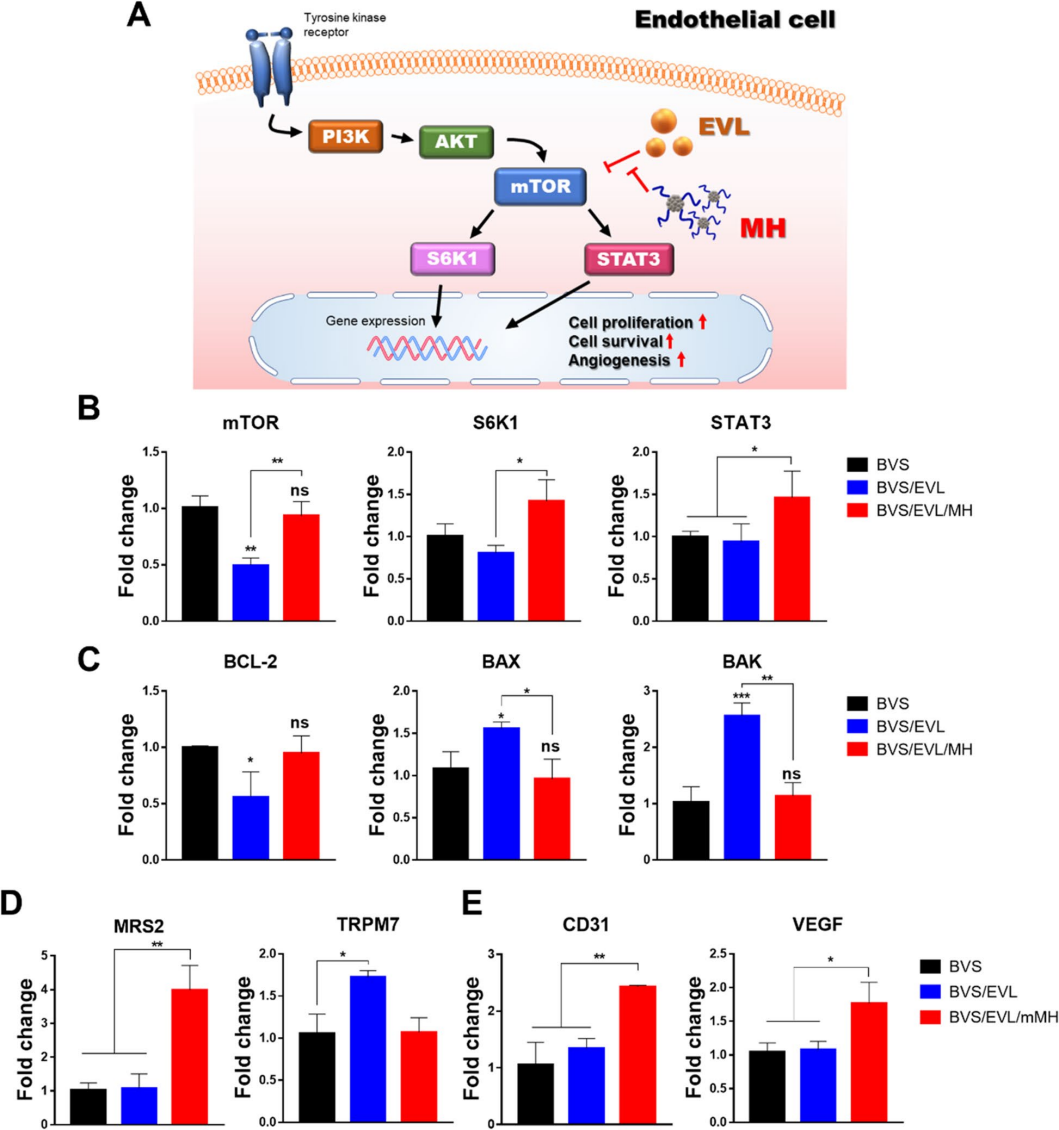
Genetic evaluation of HCAECs to confirm endothelial cell protection effects of the BVS/EVL/mMH. (**A**) Schematic illustration of proposed mechanism of the EVL and mMH effect on endothelial cell. Gene expression levels of (**B**) PI3K-AKT-mTOR pathway-, (**C**) apoptosis-, (**D**) Mg ion channel-, and (**E**) Angiogenesis-related markers on HCAECs. *p < 0.05, **p < 0.01, and ***p < 0.001 indicate statistically significant differences, respectively. PI3K: phosphoinositide-3-kinase; mTOR: mammalian target of rapamycin; S6K1: ribosomal protein S6 kinase beta-1; STAT3: signal transducer and activator of transcription 3; BCL-2: B-cell lymphoma 2; BAX: BCL-2 associated X; BAK: BCL-2 homologous antagonist killer; MRS2: mitochondrial RNA splicing protein 2; TRPM7: transient receptor potential cation channel, subfamily M, member 7; CD31: cluster of differentiation 31 (PECAM-1); VEGF: vascular endothelial growth factor

To evaluate any genetic influence of BVS/EVL/mMH, the expression of mRNA from PI3K-AKT-mTOR signaling pathway-related genes was assessed using qRT-PCR (Fig. 5A, B). Ribosomal protein S6 kinase beta-1 (S6K1) is one of the major subunits of mTOR signaling pathway that enhances protein synthesis, cell proliferation, and cell survival [31]. Signal transducer and activator of transcription 3 (STAT3) also responds to mTOR signaling to promote angiogenesis by vascular endothelial growth factor (VEGF) upregulation [32]. In the BVS/EVL/mMH group, the expression of mTOR signaling related marker mRNA was significantly alleviated compared with that in the BVS/EVL group. Furthermore, based on the ROS scavenging ability of mMH, the mRNA levels of apoptosis and Mg^2+^ channel-related mRNA levels were assessed. Expression of B-cell lymphoma 2 (BCL-2), related to anti-apoptosis, was down-regulated in the BVS/EVL (*p* < 0.05) compared with that in the BVS (Fig. 5C). However, the level of BCL-2 with BVS/EVL/mMH showed an attenuation effect on apoptosis. Expression of apoptotic markers, including BCL-2 associated X (BAX) and BCL-2 homologous antagonist killer (BAK), only increased in the BVS/EVL group. Mitochondrial RNA splicing protein 2 (MRS2) is associated with Mg^2+^ influx as a magnesium transporter into mitochondria. Transient receptor potential cation channel, subfamily M, member 7 (TRPM7) is modulated by Mg^2+^ concentration of the extracellular environment [13]. The expression of magnesium channel-related marker mRNA was regulated by existence of Mg^2+^ in the BVS/EVL/mMH (Fig. 5D). Consequently, the levels of cluster of differentiation 31 (CD31), endothelial cell marker and VEGF mRNA were remarkably upregulated in the BVS/EVL/mMH compared with that in BVS and BVS/EVL (*p* < 0.01 and *p* < 0.05) (Fig. 5E).

### Inhibition effects on smooth muscle cell of the BVS/EVL/mMH

**Fig. 6:**
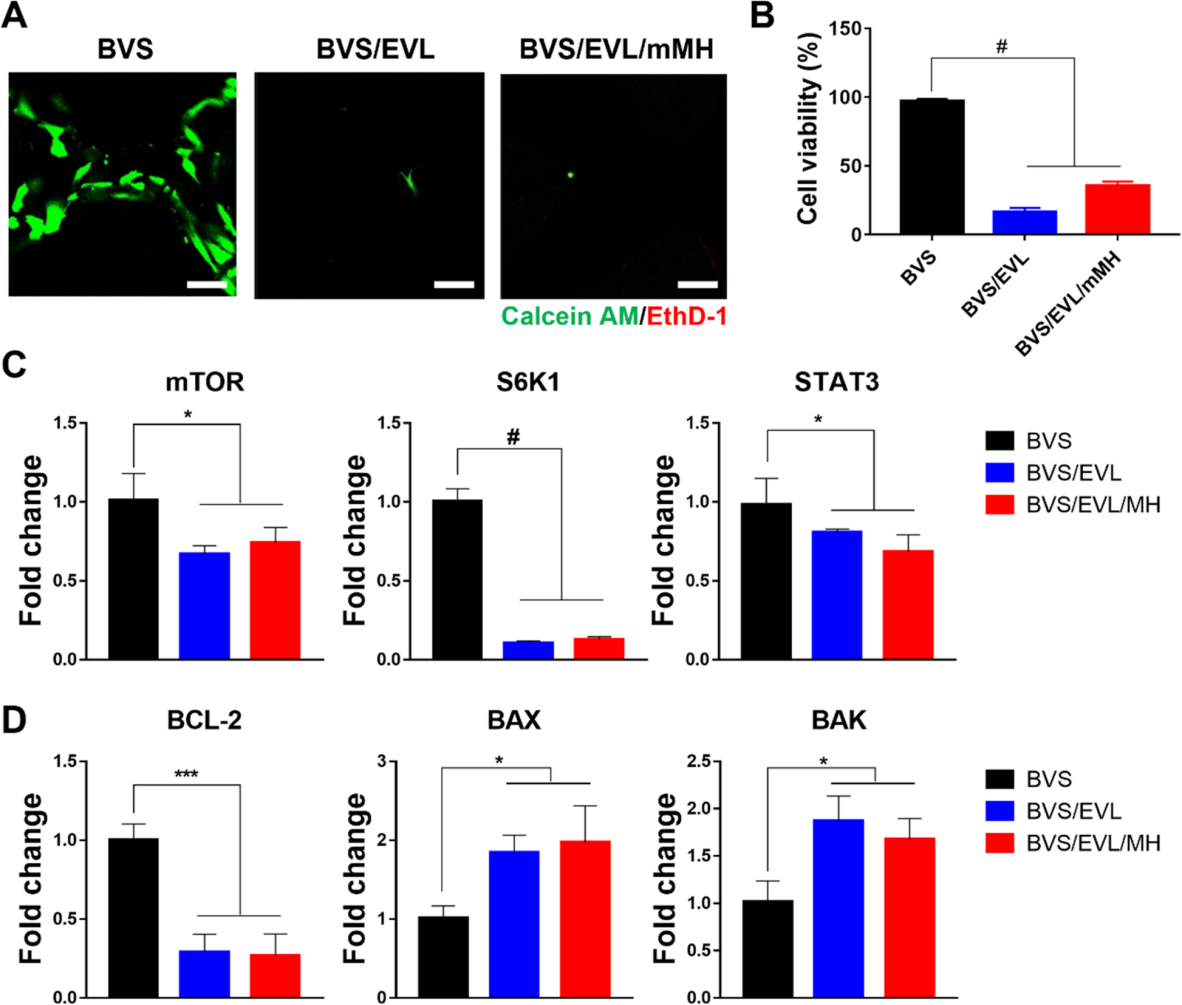
In vitro evaluation to confirm smooth muscle cell inhibition effect of the BVS/EVL/mMH. (**A**) Confocal microscopy images and (**B**) cell viability of HCASMCs cultured on the coated stents at 7 days. Scale bar indicates 100 μm. Genetic evaluation with (**C**) PI3K/AKT/mTOR- and (**D**) apoptosis-related markers of HCASMCs. *p < 0.05, **p < 0.01, ***p < 0.001, and ^#^*p* < 0.0001 indicate statistically significant differences, respectively

EVL was loaded onto BVS to reduce smooth muscle cell proliferation and thereby suppress restenosis. Based on live/dead staining images (Fig. 6A), EVL showed considerable inhibition of HCASMC viability. The quantified proliferation rate of HCASMCs notably decreased to 16.39% and 35.48% in the BVS/EVL and BVS/EVL/mMH, respectively (Fig. 6B).

To elucidate whether gene expression had been affected by the BVS/EVL/mMH, the expression of mRNA related to PI3K/ATK/mTOR signaling pathway was investigated in HCASMCs using qRT-PCR (Fig. 6C). The level of mTOR and its related markers, S6K1 and STAT3, with BVS/EVL and BVS/EVL/mMH significantly decreased (*p* < 0.05, *p* < 0.0001, and *p* < 0.05, respectively). In addition, expression of BCL-2 was remarkably down-regulated in BVS/EVL and BVS/EVL/mMH groups (*p* < 0.001) compared to that of the BVS. While expression of apoptosis-related genes including BAX and BAK increased in the BVS/EVL and BVS/EVL/mMH (Fig. 6D).

### In vivo evaluation of functionalized BVS/EVL/mMH

**Fig. 7:**
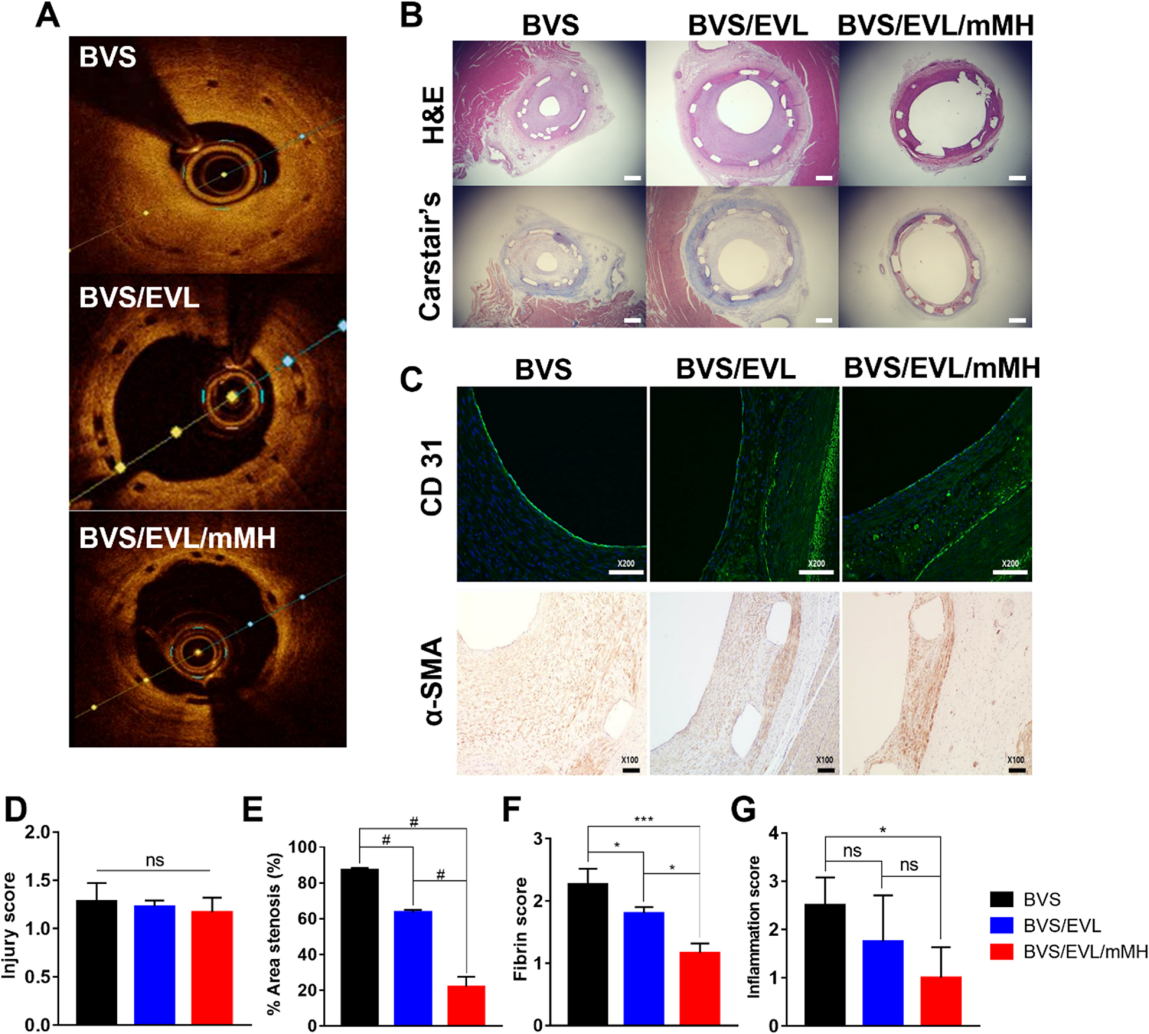
In vivo evaluation of the BVS/EVL/mMH after implantation. (**A**) Representative optical coherence tomography (OCT) images. (**B**) Histological analysis with H&E and Carstair's staining. Scale bar indicates 500 μm. (**C**) Immunohistochemistry (IHC) analysis for CD31 and SMA stained images. Quantitative biological evaluation derived from OCT and histological analysis; (**D**) injury score, (**E**) % area stenosis, (**F**) fibrin score, and (**G**) inflammation score. *p < 0.05, **p < 0.01, ***p < 0.001, and #*p* < 0.0001 indicate statistically significant differences, respectively

To evaluate the effects of the BVS/EVL/mMH on the coronary artery, this was inserted into the porcine model and observed for 4 weeks. Figure 7A shows the OCT assay images performed to identify changes in the intima area. The BVS exhibited the most severe stenosis, and there was clear narrowing of the lumen area. Occurrence of restenosis in the BVS/EVL was lower than that in BVS, and sufficient area remained in the lumen, whereas restenosis rarely occurred with BVS/EVL/mMH. Images from histological analysis using H&E and Carstair’s staining was shown in Fig. 7B. As with the OCT images, these results showed that the BVS/EVL/mMH had a larger lumen area with a reduced neointimal size compared with that found with BVS and BVS/EV. In addition, formation of thrombus around the strut of the BVS and BVS/EVL is apparent. Immunohistochemical analysis was performed to identify the expression of CD31, an endothelial cell marker, and alpha smooth muscle actin (α-SMA), a smooth muscle cell marker (Fig. 7C). Expression of CD31 was observed in all groups. However, expression of α**-**SMA was highest in the BVS and the lowest in the BVS/EVL/mMH. The quantitative biological evaluation derived from OCT and histological analysis are shown in Fig. 7D–G. There was no significant difference in injury score between the BVS groups. The area of stenosis for the BVS/EVL/mMH was lower than that for the BVS and BVS/EVL (87%, 63%, and 21% for the BVS, BVS/EVL, and BVS/EVL/mMH, respectively). BVS/EVL/mMH displayed low fibrin and inflammation scores compared to BVS and BVS/EVL.

## Discussion

This study proposed that a functional PLLA BVS coated with EVL and mMH for coronary artery disease can inhibit inflammation and restenosis and protect endothelial cells. First, we synthesized mMH developed in our previous study [19]. In FTIR analysis of the mMH, both -OH stretching vibration of MH and the carbonyl peak were observed, and the carbonyl peak was shifted because of the formation of bidentate chelating ligands [28]. The mMH maintained its function, including pH neutralization and anti-inflammation, even after modification. We comprehensively demonstrated that MH and ODLLA were combined to synthesize mMH successfully (Figs. S1, S2, S3, S4, S5). Second, we coated mMH and EVL onto BVS using an ultrasonic coater. Analyses with SEM/EDS and fluorescence imaging using FOBI showed that MH and drug were distributed on all surfaces with aggregation in BVS. These results suggest that EVL and drug releases are possible from all sites of the BVS when BVS is implanted in the body.

Since the degradation period of BVS containing PLLA is more than 2 years, proteinase K was used to evaluate the accelerated degradation behavior over 7 days. Proteinase K can degrade PLLA because the monomer of PLLA has a structure similar to alanine [33]. During degradation, the pH of the BVS decreased to 5.77. The pH value hydrolyzed at 37 °C of PLLA was reported to maintain neutral for up to 40 weeks, whereas our BVS degraded using proteinase K was neutral for only 2 days [34, 35]. In comparison, the 7 days in our degradation system is expected to equivalent to degradation at 37 °C for approximately 3 years. The BVS/EVL/mMH maintained a near-neutral pH during the 7 days accelerated degradation period, indicating that this can neutralize byproducts for 3 years after implantation in the body. Even after degradation for 7 days, 77.27% of MH remained in BVS/EVL/mMH, which may maintain the neutralizing effect until the BVS is completely degraded. EVL release, measured in PBS solution at 37 °C in an environment similar to the human body can inhibit HCASMC viability for one month after which reduced release is not expected to interfere with re-endothelialization [30].

To examine the occurrence of thrombosis when a stent is implanted into the body, protein adsorption and platelet adhesion were measured. Protein adsorption is the first reaction that occurs in blood when in contact with biomaterials [36]. This was performed using fibrinogen and albumin as the major proteins in the blood. Fibrinogen selectively adsorbs on the hydrophobic surface and induces platelet adhesion [37]. Although mMH is surface-modified with a hydrophobic oligomer, the intrinsic hydrophilic property of MH had a lower water contact angle than that of PLLA [28]. The mMH in the BVS/EVL/mMH leads to more hydrophilicity than was induced by the BVS/EVL and inhibited fibrinogen adsorption. Albumin passivates the surface of biomaterials and decreases platelet adhesion and aggregation [38]. Thus, increased albumin adsorption in BVS/EVL/mMH could inhibit thrombosis. The protein adsorption was estimated using AFR, and the BVS/EVL/mMH showed a higher AFR than either BVS or BVS/EVL. This suggests that the albumin may be adsorbed more than fibrinogen was, thereby inhibiting platelet adhesion. The adhesion and activity of platelets following evaluation of protein adsorption were investigated and showed that platelet adhesion and activation of the BVS/EVL and BVS/EVL/mMH were reduced. Moore et al*.* reported that thrombin stimulates mTORC1 activation via protein kinases C and a P2Y_12_-stimulated pathway, leading to platelet adhesion and activation [39]. EVL inhibits thrombin-induced mTORC1 activation to suppress platelet adhesion and aggregation. In addition, hydrophilicity increased by MH also inhibits platelet adhesion and activation. Therefore, the synergistic effect of EVL and mMH effectively inhibited protein adsorption and platelet adhesion, demonstrating that the BVS/EVL/mMH is compatible with blood.

Because of established biocompatibility of PLLA, great adhesion and viability of HCAECs initially on the BVS were observed (Fig. 4A, B). Based on wound healing analysis, the BVS/EVL group seldom displayed cell migration because of strong inhibition effect of EVL on cellular metabolism. Interestingly, the mMH incorporated group exhibited outstanding cell migration similar to that of the control. As illustrated in Fig. 5A, mMH could attenuate the inhibition ability of PI3K/Akt/mTOR signal induced by EVL thereby encouraging endothelial cell protection. Furthermore, MH facilitates ROS scavenging to enhance endothelial cell viability [40, 41]. The released free Mg^2+^ ions from mMH can play as a radical scavenger for the hydroxyl radical and superoxide anions released by PLLA [13, 42]. Therefore, the representative DCF-DA stained image of BVS/EVL/mMH was observed to lower fluorescence intensity compared with that of BVS and BVS/EVL.

As mentioned above, EVL is a key inhibitor of the PI3K-AKT-mTOR pathway in mammalian cells. When the EVL is utilized as a restenosis reducer for stent, the crucial concern is the simultaneous occurrence of endothelial cell damage. Thus, we focused on whether mMH could alleviate the EVL-induced cytotoxicity on endothelial cells. Activated mTORC1 phosphorylates S6K1, which then phosphorylates multiple substrates, including eukaryotic translation elongation factor 2 kinase, eukaryotic elongation factor 2 kinase, eukaryotic translation initiation factor 4B, programmed cell death 4, and adenosine monophosphate. Consequently, these activated genes could enhance protein synthesis, cell growth, and cell proliferation [31] (Fig. 5A). STAT3 is activated by phosphorylation in response to various proteins, including mTOR, IFNs, EGF, and BMP2. Phosphorylated STAT3 plays a key role in many cellular processes such as cell growth, apoptosis, and angiogenesis [43]. Assessment of mRNA expression in HCAECs exposed to BVS/EVL/mMH implied that mMH could reduce endothelial cell damage induced by EVL (Fig. 5B). Expression of anti-apoptotic, magnesium ion channel, and endothelial cell marker mRNA also demonstrated the versatile capacity of mMH (Fig. 5C–E).

Biological activity of the multifunctional BVS/EVL/mMH on HCASMCs was directly evaluated relating restenosis. Despite the presence of mMH, the viability of HCASMCs were completely inhibited (Fig. 6A, B). This suggests that mMH could only selectively protect HCAECs. An inhibitory effect of HCASMC by EVL, same as the previous evaluation with HCAECs, was demonstrated with the PI3K/ATK/mTOR signaling pathway. Because of suppressed mTORC1 only in smooth muscle cells, also phosphorylation of S6K1 and STAT3 was inhibited [29]. Consequently, cell proliferation was reduced, and apoptosis was induced (Fig. 6D).

The BVS/EVL/mMH was inserted into porcine coronary arteries to confirm the clinical function (Fig. 7). Inflammatory response and neointima formation were induced by acidic degradation byproducts of BVS and BVS/EVL. However, the inflammatory response of the BVS/EVL/mMH was reduced by neutralizing effect of mMH [26, 27]. In addition, BVS/EVL/mMH synergistically inhibited smooth muscle cell proliferation and protected endothelial cells producing a low restenosis ratio in the coronary arteries. Thus, the BVS/EVL/mMH showed superior function as a stent.

## Conclusion

Although the first-generation BVS, Absorb™ BVS, was reported as a clinical failure process, the BVS is still highly evaluated as a cardiovascular stent because of the biodegradability. However, problems with BVS such as restenosis, inflammation caused by degradation products, and neointimal formation need to be resolved. In this study, we proposed a functional PLLA BVS coated with EVL and mMH for treatment of coronary artery disease. Protein adsorption and platelet adhesion were induced by EVL and reduced by mMH, indicating that our developed BVS has excellent blood compatibility. The BVS/EVL/mMH selectively inhibited inflammatory response and restenosis by suppression of smooth muscle cell proliferation and enhanced re-endothelialization by protected endothelial cells in vitro and in vivo. Such a functional PLLA BVS would be expected as an alternative to DES and the existing BVS for cardiovascular diseases.

## Supplementary Information

**Additional file 1:** **Fig. S1. **TEM image of mMH. Scale bar: 100 nm. **Fig. S2.** Size distribution of mMH. **Fig. S3. **ATR-FTIR spectra of MH, DL-Lactide, and mMH. **Fig. S4. **TGA thermograms of MH and mMH. **Fig. S5. **Neutralizing effect of MH and mMH. **Fig. S6. **Dispersion stability of coating solution with unmodified MH and mMH. **Fig. S7. **SEM images and EDS mapping of C, O, and Mg elements of the BVS, and BVS/EVL. Scale bar: 5 μm. **Fig. S8. **XRD patterns of the PLLA, MH, BVS, BVS/EVL, and BVS/EVL/mMH. **Fig. S9. **Mechanical properties of the BVS, BVS/EVL, and BVS/EVL/mMH. (A) tensile strength, (B) elongation, and (C) Young’s modulus. **Table S1. In vitro **qRT-PCR primer sequences.

## Data Availability

All data is available upon request to the corresponding author.

## Abbreviations

PLGA: Poly(lactic-co-glycolic) acid
EVL: Everolimus
mMH: Modified MH with ODLLA
ODLLA: Oligo-D,L-lactide
MH: Magnesium hydroxide
CAD: Coronary arteries disease
BMS: Bare metal stent
Co-Cr: Cobalt chromium
SS: Stainless steel
ISR: In-stent restenosis
DES: Drug-eluting stent
PLLA: Poly(L-lactic acid)
mTOR: Mammalian target of rapamycin
FRB: FKBP12-rapamycin-binding
CF: Chloroform
THF: Tetrahydrofuran
HCAECs: Human coronary artery endothelial cells
HCASMCs: Human coronary artery smooth muscle cells
EGM-2: Endothelial cell growth medium-2
SmGM: Smooth muscle cell growth medium
DPBS: Dulbecco’s phosphate buffered saline
CCK-8: Cell-counting kit 8
DCF-DA: 2′7’-Dichlorodihydrofluorescein
TEM: Transmission electron microscopy
ATR-FTIR: Attenuated total reflection-Fourier transform infrared
TGA: Thermogravimetric analyzer
DLS: Dynamic laser scattering
FE-SEM: Field emission-scanning electron microscopy
EDS: Energy dispersive spectroscopy
XRD: X-ray diffractometer
HPLC: High Performance Liquid Chromatography
ICP-OES: Inductively Couples Plasma Optical Emission Spectrometer
WAD: Weight after degradation
WBD: Weight before degradation
PRP: Platelet-rich plasma
PPP: Platelet-poor plasma
LDH: Lactose dehydrogenase
OCT: Optical coherence tomography
TTC: 2,3,5-Triphenyl tetrazolium chloride
SMA: Smooth muscle actin
H&E Staining: Hematoxylin and eosin staining
NR: Nile Red
UTM: Universal testing machine
EthD-1: Ethidium homodimer-1
ROS: Reactive oxygen species
S6K1: S6 kinase beta-1
STAT3: Signal transducer and activator of transcription 3
BCL-2: B-cell lymphoma 2
BAX: Bcl-2 associated X
BAK: Bcl-2 homologous antagonist killer
MRS2: Mitochondrial RNA splicing protein 2
TRPM7: Transient receptor potential cation channel, subfamily M, member 7
CD31: Cluster of differentiation 31
VEGF: Vascular endothelial growth factor
H&E: Hematoxylin and eosin
