# Supplementary material for: Reduced restenosis and enhanced re-endothelialization of functional biodegradable vascular scaffolds by everolimus and magnesium hydroxide
Source: Biomater Res. 2022 Dec 21;26:86. doi: 10.1186/s40824-022-00334-x (PMC9768885; doi:10.1186/s40824-022-00334-x)
Supplement: Supplementary file 1 — Additional file 1: Fig. S1. TEM image of mMH. Scale bar: 100 nm. Fig. S2. Size distribution of mMH. Fig. S3. ATR-FTIR spectra of MH, DL-Lactide, and mMH. Fig. S4. TGA thermograms of MH and mMH. Fig. S5. Neutralizing effect of MH and mMH. Fig. S6. Dispersion stability of coating solution with unmodified MH and mMH. Fig. S7. SEM images and EDS mapping of C, O, and Mg elements of the BVS, and BVS/EVL. Scale bar: 5 μm. Fig. S8. XRD patterns of the PLLA, MH, BVS, BVS/EVL, and BVS/EVL/mMH. Fig. S9. Mechanical properties of the BVS, BVS/EVL, and BVS/EVL/mMH. (A) tensile strength, (B) elongation, and (C) Young’s modulus. Table S1. In vitro qRT-PCR primer sequences. [file 40824_2022_334_MOESM1_ESM.docx]

Supplementary Information

**Suppression of Smooth Muscle Cells and Protection of Endothelial Cells on Functional Biodegradable Vascular Scaffolds**

Seung-Woon Baek^1,2,3,†^, Duck Hyun Song^1,†^, Da-Seul Kim^1,4†^, Han Byul Kim^5^, Semi Lee^1^, Jun Hyuk Kim^1^, Young Joon Hong^6^, Chun Gwon Park^2,3^ and Dong Keun Han^1^,*

^1^ Department of Biomedical Science, CHA University, 335 Pangyo-ro, Bundang-gu, Seongnam-si, Gyeonggi-do 13488, Korea;

^2^ Department of Biomedical Engineering, SKKU Institute for Convergence, Sungkyunkwan University (SKKU), 2066 Seobu-ro, Jangan-gu, Suwon-si, Gyeonggi-do 16419, Korea;

^3^ Department of Intelligent Precision Healthcare Convergence, SKKU Institute for Convergence, Sungkyunkwan University (SKKU), 2066 Seobu-ro, Jangan-gu, Suwon-si, Gyeonggi-do 16419, Korea

^4^ School of Integrative Engineering, Chung-Ang University, 84 Heukseok-ro, Dongjak-gu, Seoul 06974, Korea;

^5^ Division of Biotechnology, College of Life Sciences and Biotechnology, Korea University, Seoul 02841, Korea

* Correspondence: dkhan@cha.ac.kr (D.K.H.)

† These authors contributed equally to this work.


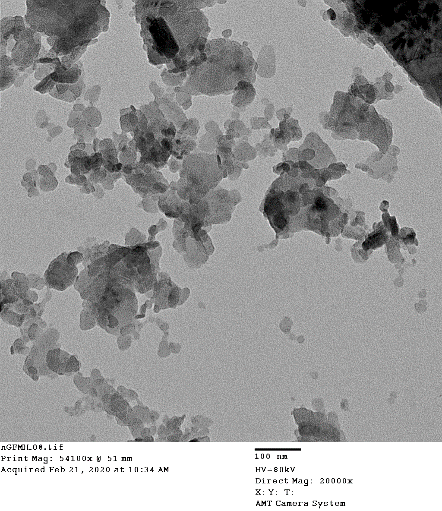


**Fig. S1** TEM image of mMH. Scale bar: 100 nm.

**Fig. S2** Size distribution of mMH.

**
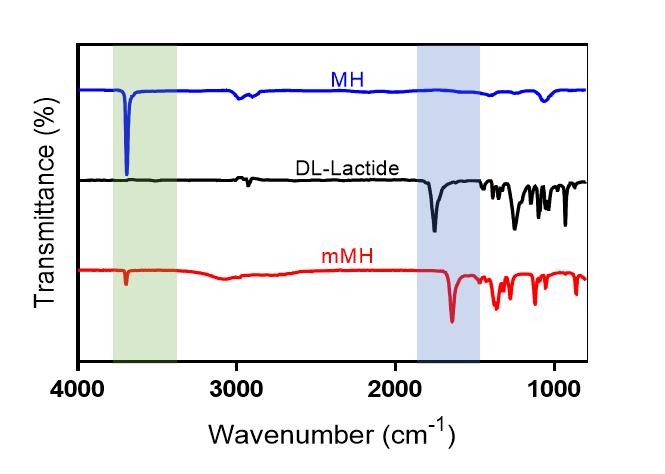
**

**Fig. S3** ATR-FTIR spectra of MH, DL-Lactide, and mMH

**Fig. S4** TGA thermograms of MH and mMH.

**Fig. S5** Neutralizing effect of MH and mMH.


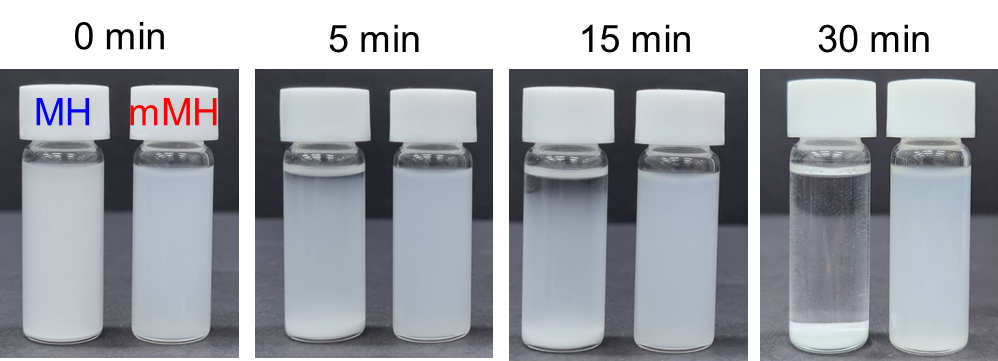


**Fig. S6** Dispersion stability of coating solution with unmodified MH and mMH.


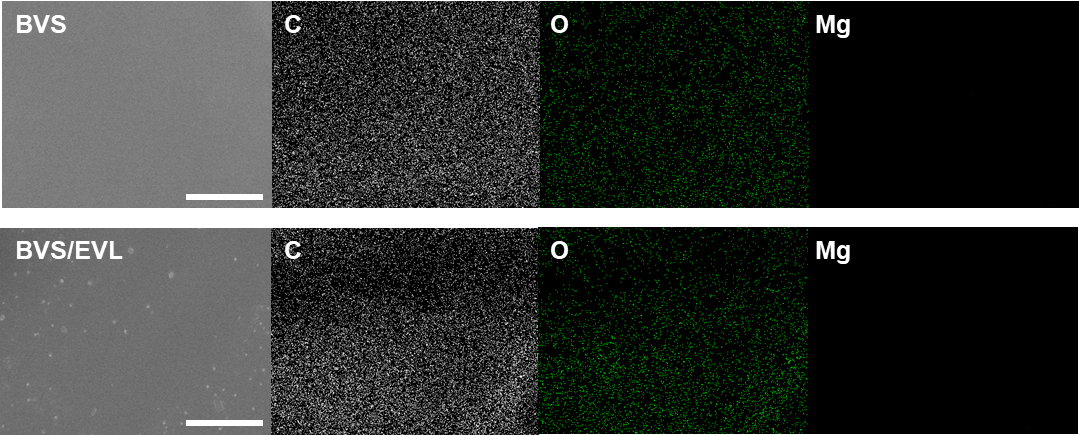


**Fig. S7** SEM images and EDS mapping of C, O, and Mg elements of the BVS, and BVS/EVL. Scale bar: 5 μm.

**Fig. S8** XRD patterns of the PLLA, MH, BVS, BVS/EVL, and BVS/EVL/mMH.


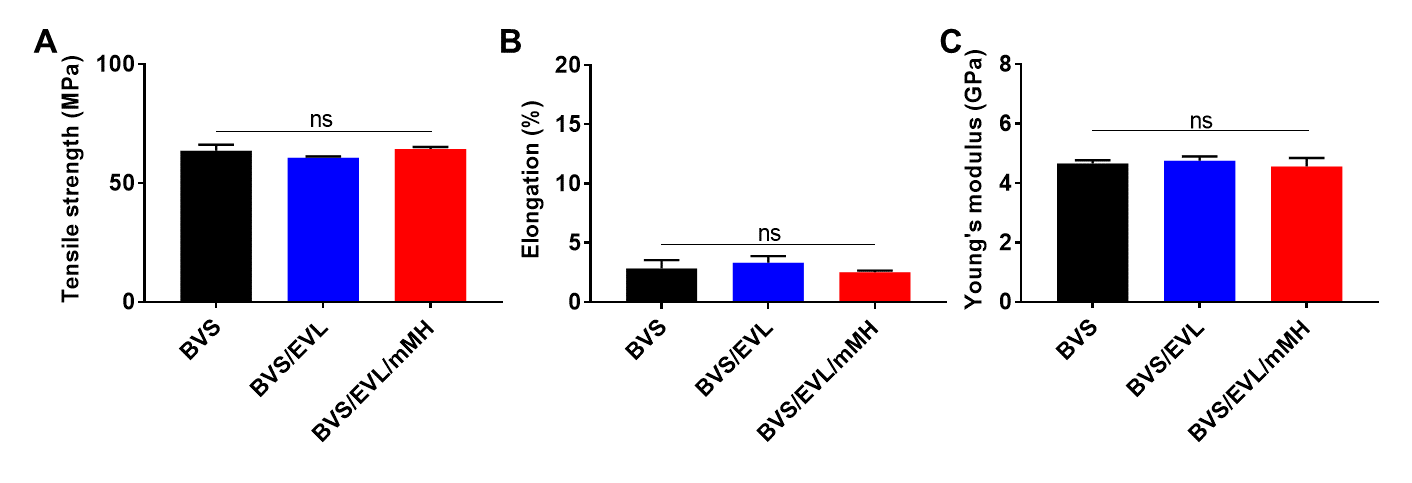


**Fig. S9** Mechanical properties of the BVS, BVS/EVL, and BVS/EVL/mMH. (A) tensile strength, (B) elongation, and (C) Young’s modulus.

**Table S1.** *In vitro* qRT-PCR primer sequences

| Gene | Forward | Reverse |
| --- | --- | --- |
| 18S | GCAATTATTCCCCATGAACG | GGGACTTAATCAACGCAAGC |
| mTOR | TCCGAGAGATGAGTCAAGAGG | CACCTTCCACTCCTATGAGGC |
| S6K1 | AGAACTTCTGGCTCGAAAGGT | CGACAGGTGTCTGACGTGTAA |
| STAT3 | CCCTTGGATTGAGAGTCAAG | AAGCGGCTATACTGCTGGTC |
| BCL-2 | AGTACCTGAACCGGCACCT | GCCGTACAGTTCCACAAAGG |
| BAX | CATCATGGGCTGGACATTG | GGGACATCAGTCGCTTCAGT |
| BAK | AGACCTGAAAAATGGCTTCG | CGGAAAACCTCCTCTGTGTC |
| MRS2 | CCAGAACCATGGAATGCCTGC | GTATCCACGGGGCTGTTCCTAAC |
| TRPM7 | TTGACATTGCCAAAAATCATGT | CTTGTTCCAAGGATCCAACC |
| CD31 | GCAACACAGTCCAGATAGTCGT | GACCTCAAACTGGGCATCAT |
| VEGF | ACTGGACCCTGGCTTTACTG | TCTGCTCCCCTTCTGTCGT |
